# Ready for change: Oscillatory mechanisms of proactive motor control

**DOI:** 10.1371/journal.pone.0196855

**Published:** 2018-05-16

**Authors:** Matthias Liebrand, Jascha Kristek, Elinor Tzvi, Ulrike M. Krämer

**Affiliations:** 1 Department of Neurology, University of Lübeck, Lübeck, Germany; 2 Graduate School for Computing in Medicine and Life Sciences, University of Lübeck, Lübeck, Germany; 3 Institute of Psychology II, University of Lübeck, Lübeck, Germany; Universita degli Studi di Roma La Sapienza, ITALY

## Abstract

Proactive motor control is a preparatory mechanism facilitating upcoming action inhibition or adaptation. Previous studies investigating proactive motor control mostly focused on response inhibition, as in the classical go-nogo or stop-signal tasks. However, everyday life rarely calls for the complete suppression of actions without subsequent behavioral adjustment. Therefore, we conducted a modified cued go-nogo-change task, in which cues indicated whether participants might have to change to an alternative action or inhibit the response to an upcoming target. Based on the dual-mechanisms of control framework and using electroencephalography (EEG), we investigated the role of the sensorimotor cortex and of prefrontal regions in preparing to change and cancel motor responses. We focused on mu and beta power over sensorimotor cortex ipsi- and contralateral to an automatic motor response and on prefrontal beta power. Over ipsilateral sensorimotor cortex, mu and beta power was relatively decreased when anticipating to change or inhibit the automatic motor behavior. Moreover, alpha phase coupling between ipsilateral motor cortex and prefrontal areas decreased when preparing to change, suggesting a decoupling of sensorimotor regions from prefrontal control. When the standard motor action actually had to be changed, prefrontal beta power increased, reflecting enhanced cognitive control. Our data highlight the role of the ipsilateral motor cortex in preparing to inhibit and change upcoming motor actions. Here, especially mu power and phase coupling seem to be critical to guide upcoming behavior.

## Introduction

“Change is inevitable. Change is constant.” (Benjamin Disraeli, 1867).

A key human feature is the ability to adapt to an ever-changing environment. This capability requires cognitive control, namely the ability to flexibly change or cancel preexisting action plans. Situations differ though in the likelihood of relevant changes to happen, for instance when driving on a street having right of way or when approaching traffic lights which might change. This study concentrates on motor control, one aspect of cognitive control. Most motor control studies focused on the neural response after changes occur, i.e. so-called reactive motor control. Even more important might be, though, how we prepare for upcoming changes in terms of biasing perceptual processes or motor preparation, which is termed proactive motor control. Here, we studied proactive motor control using electroencephalography (EEG) in a cued reaction time task.

The dual mechanisms of control (DMC) framework by Braver states that cognitive control can be employed in a proactive or in a reactive way, depending on current costs and benefits of the respective processes [1, 2]. It hypothesizes that proactive control is based on prefrontal activity, attention and modulation of activity in a motor network. Perceptual and action systems are thought to be biased in a goal-driven manner. In a recent study using EEG in a cued go/nogo paradigm, we investigated the interplay of prefrontal cognitive control, visual attention and sensorimotor activity mediating proactive motor inhibition [3]. We contrasted trials in which a cue signaled that an upcoming motor action might have to be inhibited with others where no cancellation was necessary. During the cue-target interval, we found that the anticipation of nogo-signals led to increased visual attention, reflected in reduced occipital alpha power, and to a modulation of sensorimotor cortex activity, reflected in reduced beta power ipsilateral to the relevant response hand. Prefrontal activity, indicated by increased beta power, was observed only after target presentation, but not during the cue-target interval. We interpreted the increase in prefrontal beta power as enhanced cognitive control. In the present study, we focused on sensorimotor and prefrontal mechanisms, given their principal role in motor inhibition. Attentional effects were replicated, but did not provide novel insights into stopping processes and thus are not reported to prevent overextensive results.

Prefrontal cortex and specifically prefrontal beta oscillations have previously been implicated in proactive and reactive motor inhibition [4, 5]. Studies investigating reactive inhibition reported increased prefrontal beta power when subjects had to inhibit a motor response [6, 7]. Together with evidence of basal ganglia dependent changes in cortical beta oscillations [8–10], these observations led to the hypothesis of an inhibition-related cortico-basal ganglia network mediated by beta activity [4]. For proactive motor inhibition a similar prefrontal mechanism seems to be relevant as in reactive control. For instance, beta power over right dorsolateral prefrontal cortex (dlPFC) increased before successfully inhibiting a saccade [11]. Two studies using a modified stop signal task (SST) in a small group of epileptic patients found evidence for right inferior frontal cortex (IFC) and dorsolateral prefrontal cortex (PFC) to be implicated in proactive inhibition, there indicated by increased gamma (and not beta) power over those regions [12, 13]. Importantly, in these two studies prefrontal regions were activated only after, but not before target signals. Even though counterintuitive, proactive effects can take place after appearance of target-signals. Specifically, having anticipated a possible inhibition modulated target-related processing but not activity in the cue-target interval. This, along with our previous finding of target-related prefrontal beta activity suggests that prefrontal beta activity provides a phasic control signal in response to the target. Prefrontal beta seems to play less of a role in a sustained way during the preparatory period between cue and target.

Over sensorimotor cortex, both mu and beta power decrease during movement preparation [14, 15] with a stronger decrease contralateral to the expected action [16, 17]. In general, beta power has been linked to motor cortex excitability, as measured by transcranial magnetic stimulation (TMS) and motor evoked potentials (MEP) [18]. In a recent study, Tzagarakis, West [19] investigated motor preparation in terms of directional uncertainty of an upcoming movement. In the task, the direction in which a joystick had to be pulled was cued to be at a specific location or within a range of 90° or 180°. The authors investigated beta power over sensorimotor cortex in the interval between cue and target. They found that the greater the directional certainty of a movement, the lower the beta power, and thus concluded that beta power reflects the level of motor preparation. In another study, in which a wrist extension was signaled by sounds, the pre-movement decrease of central beta power was similarly interpreted to reflect preparation of movements [6]. Our finding of a stronger beta power decrease ipsilateral to the relevant response hand when anticipating an inhibition [3] supports the significance of beta in motor planning. Moreover, it suggests that the activation of the ipsilateral motor cortex or activity differences between ipsi- and contralateral motor cortex are important for motor preparation. Also sensorimotor mu power, and not beta alone, seems to be relevant for proactive motor control. Prestimulus mu has been shown to be elevated before commission errors in a go-nogo task, indicating that these errors might result from decreased motor cortex excitability already before target presentation [20].

Prefrontal and sensorimotor cortex can be assumed to form a network, probably involving the basal ganglia [4], mediating inhibitory motor control. This asks for a network approach to characterize neural dynamics and connectivity changes related to proactive motor control. A putative marker for information flow within neural networks is phase-coupling [21]. During recent years, phase synchrony has increasingly attracted attention as a measure of both local computations [22] and largescale integration [21], subserving cognitive functions as attention [23], motor control [24, 25] or memory [26]. It has been shown that interhemispheric fronto-frontal and intrahemispheric fronto-central theta and alpha phase coupling is increased when motor output has to be inhibited [27–29]. Moreover, alpha coupling is aberrant in patients with movement disorders, such as musician’s dystonia [27] and Parkinson’s disease [30], but also Tourette’s syndrome [31]. Also, prestimulus alpha coupling seems to have an impact on behavior, as interhemispheric coupling correlated negatively with reaction times in a go-nogo-task [32].

To sum up, converging evidence from invasive and noninvasive EEG-studies speaks for specific modulations of sensorimotor mu/beta and prefrontal beta oscillations when pro- or reactively inhibiting motor output. Critically, most studies on proactive and reactive motor control focused on motor inhibition using variants of the classical go/nogo and stop-signal tasks. In these studies a prepotent standard motor action has to be cancelled when nogo- or stop-signals appear. This has its limitations though, since everyday life rarely calls for the complete suppression of actions but rather for behavioral adjustments and execution of alternative actions (for review see Böcker, Gauggel [33]). One action has often to be inhibited (e.g. pushing the gas pedal), while another action is executed at the same time (e.g. pushing the brake). This can be studied with change-stop tasks [34], as for instance the change-signal paradigm [35, 36]. In those tasks, in change-trials a frequent motor action has to be inhibited and replaced with an alternative motor response. However, there is sparse knowledge about the neural network dynamics underlying the preparation to change.

Extending our previous results, we were interested in what role sensorimotor mu and beta play in preparing to switch actions and as to whether the inhibition-related prefrontal beta effects generalize to action change. We developed a cued go-nogo-change task, in which cues indicated that participants might have to inhibit the standard go response (button press with one hand), might have to switch to a different response (button press with the other hand) or have to give the standard response with certainty.

Based on the DMC framework, above-mentioned findings and our previous study, we focused on oscillatory activity (mu/beta) over sensorimotor and prefrontal regions and on network interactions. For the cue-target interval, when preparing to possibly inhibit or change one’s action, we expected a decrease of ipsilateral mu/beta replicating and extending our previous study. We hypothesized prefrontal beta to increase after target presentation, both if the standard action had to be inhibited or changed. As increased alpha phase coupling between prefrontal and central sites has been observed when actually inhibiting a motor response [27], we expected similarly increased connectivity when preparing to adapt one’s action.

## Methods

### Subjects

Twenty-eight participants took part in the study. One participant was excluded due to mixed-handedness and five participants had to be excluded due to extensive EEG artifacts (further explanation given below). All of the remaining 22 participants (20–32 years, mean: 24.8 years, 15 women) were right-handed and by self-report free of neurological or psychiatric disorders. The participants had normal or corrected to normal vision. They gave informed consent and received money (8€/h) or course credit for participation. The study was performed in agreement with the Declaration of Helsinki and had been approved by the ethics committee of the University of Lübeck.

### Design and stimuli

**Fig 1:**
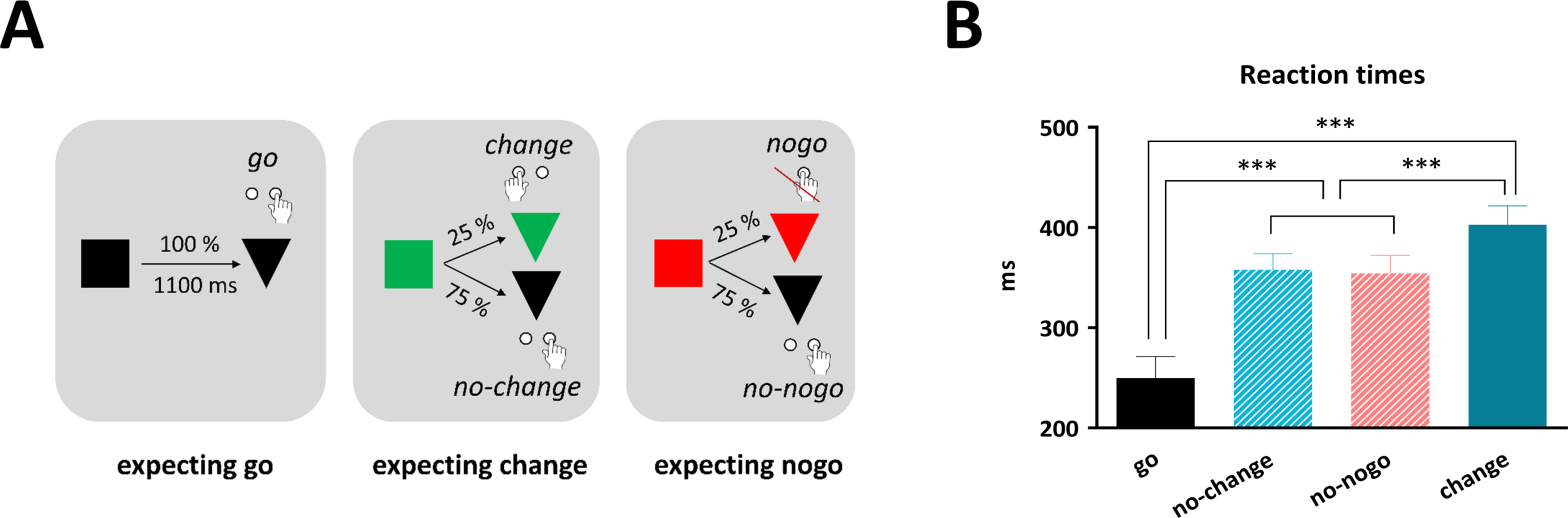
Design and behavioral results. (A) Design of the cued go-nogo-change task. In expecting go-trials, the cue (black square) was always followed by a black triangle, indicating a right button press. In expecting change-trials, the cue (green square) was followed in 75% by a black triangle, indicating a right button press and in 25% by a green triangle, indicating a left button press. In expecting nogo-trials, the cue (red square) was followed in 75% by a black triangle, indicating a right button press and in 25% by a red triangle, indicating no button press. In the second half of the experiment, the matching of the response buttons was reversed (meaning expecting go-trials required a left button press etc.). (B) Reaction times. Mean reaction times in ms in go-, no-change-, no-nogo- and change-trials. As error bars the standard error of the mean (SEM) is depicted. Participants responded faster in go- than no-change-/no-nogo- than change-trials. Significant effects are indicated with asterisks (* for ≤ 0.05, ** for ≤ 0.01, *** for ≤ 0.001).

The participants performed in a cued go-nogo-change-task (Fig 1). The experiment included three different conditions: expecting go (EG), expecting change (EC), and expecting nogo (EN). Each condition occurred with the same probability (33%). In all trials, a cue (square) appeared first, indicating the condition, followed by a target (triangle) which signaled the action to be performed. In expecting go-trials, a black square appeared first, which was always followed by a black triangle. The participants were instructed to press the right button with their right index finger once the triangle had appeared (go condition). Expecting change-trials began with a green square, followed either by a black or a green triangle. If the green square was followed by a black triangle (75% of the expecting change-trials), the participant had to press the right button (no-change condition). Once the green square was followed by a green triangle (25% of the expecting change-trials), the participant was supposed to press the left button with the left index finger (change condition). Expecting nogo-trials started with a red square, followed either by a black or a red triangle. In case the red square was followed by a black triangle (75% of the expecting nogo-trials), the participant had to press the right button (no-nogo condition). Once the red square was followed by a red triangle (25% of the expecting nogo-trials), the participant was supposed to refrain from pressing any button (nogo condition). In no-change- and no-nogo-trials, participants thus were prepared to cancel or change the motor response, but had to execute the standard action finally. In nogo- and change-trials, participants actually had to switch their standard behavior to an alternative action or to no action. Our choice for a low percentage of nogo-trials is in line with a recent study, suggesting that fast-paced go/nogo tasks with low share of nogo-trials are best suited to capture activity actually related to motor inhibition [37].

The cue (square) was presented for 100 ms. After an inter-stimulus interval of 1 s, the target (triangle) followed, which was also shown for 100 ms. The time between two subsequent trials was jittered (1.3 to 1.6 s). The experiment was divided into 6 blocks with 160 trials each, resulting in 960 trials. After half of the experiment (3 blocks), the assignment of the response hands was switched, meaning that participants were instructed to press the left button according to the black triangle and the right button in reaction to the green triangle (left handed blocks, LHB). The assignments of response hands of the first and second half were counterbalanced among participants. Half of the participants started with three blocks in which right handed actions were standard (right handed blocks, RHB), the other half with three blocks, in which left handed actions were standard (LHB). Before the start of the experiment, the participants practiced the task in two short blocks with 12 trials each, using both hands as standard actions. Throughout the whole experiment, a line was presented underneath the stimuli, which participants were instructed to fixate. Participants were told to respond as fast and accurately as possible and not to press the button until the triangle had appeared.

### Procedure

The experiment was controlled using the Presentation® software (Version 14.5). Stimuli were presented on a 17” screen, about 1 m away from the participant. Participants were sitting in a comfortable chair. In the middle and at the end of each block they had a short break of 20 s to relax. The total duration of the experiment was about 50 min.

### EEG recordings and data preprocessing

The EEG was recorded with a 64-channel BrainAmp MR plus amplifier with a sampling rate of 250 Hz, resolution of 0.1 μV and a 0.1–250 Hz pass band. Electrodes were placed according to an extension of the international 10–20 system [38]. Vertical and horizontal eye movements (vEOG and hEOG) were recorded, the former using an electrode placed below the right eye and a frontopolar electrode, the latter using electrodes located on the outer canthus of each eye. The EEG was recorded against a reference electrode placed on the right earlobe.

### Behavioral data analyses

Mean reaction times, hit rates and premature responses were computed for each subject and submitted to repeated measures ANOVAs with the within-subject factor Condition (go, no-change, change, no-nogo) and to subsequent paired-sample t-tests.

### EEG data analyses

EEG data analysis was performed with EEGLAB [39] and custom written MATLAB (Natick, MA) scripts. EEG data were re-referenced offline to the average of the signal from the two earlobe electrodes. The data were high-pass filtered with 0.5 Hz in addition to a notch filter of 50 Hz. The data were segmented into epochs for the different conditions. Epochs included 1 s before and 2 s after the stimulus. The baseline was defined as the 100 ms preceding the stimulus. An Independent Components Analysis (ICA) as implemented in EEGLAB (Infomax extended), was performed on the segmented data including all conditions. Independent components accounting for blink artifacts and horizontal eye movements were visually identified and removed from the data (mean 2.7, range: 1–4 per subject) [40]. Additionally, trials affected by other artifacts caused e.g. by muscle tension were rejected from further analysis using an amplitude threshold rejection of ±80 μV. If more than 30% of the data of one participant were rejected, this subject was excluded from analysis (five subjects). Current source density interpolation of the data was estimated through a spatial Laplacian transform based on a spherical spline interpolation (using a spline order of 4) [41] using a current density toolbox for MATLAB [42]. We took advantage of the Laplace transformation, as it improves the spatial resolution of EEG, especially in combination with high density recordings (≥ 64 electrodes) [43].

### Time-frequency analysis

To study the inhibition related power changes in the alpha (9–14 Hz) and beta band (15–25 Hz), single trial data were convolved with a complex Morlet wavelet as implemented in MATLAB (function cwt with parameter specification ‘cmor1-1.5’):

$$w(t)={\left(\pi f_{b}\right)}^{-0.5}e^{-2\pi i{ft}_{c}}e^{-\frac{t^{2}}{f_{b}}}$$

Where *f_b_* = 1 was the bandwidth parameter and *f_c_* = 1.5 was the wavelet center frequency [44]. Specifically, we computed and averaged for each subject, changes in time varying energy (square of the convolution between wavelet and signal) in the studied frequencies (1–40 Hz, linear increase) with respect to a pre-stimulus baseline (-250 to -50 ms prior to the stimulus). The selection of the analyzed alpha/mu (9–14 Hz) and beta (15–25 Hz) frequencies was based on visual inspection of the data and previous literature [3, 36, 45]. In order to reduce the number of statistical comparisons and to increase the signal-to-noise ratio, we clustered the electrodes into regions of interest: Left prefrontal (F3, F5, FC3, FC5), right prefrontal (F4, F6, FC4, FC6), left central (C3, C5, CP3, CP5) and right central (C4, C6, CP4, CP6), based on previous studies [3, 25].

Effects over prefrontal and sensorimotor electrodes were analyzed differently. For motor related effects (sensorimotor mu and beta) data of left handed blocks (LHB) were flipped along the midline. This allowed for analysis of effects dependent on the side (contra- vs. ipsilateral) relative to the response hand. For prefrontal effects (beta power) no lateralization relative to the response hand was expected. Thus data were not flipped and we compared data over left and right hemisphere. For all effects, trials of RHB and LHB blocks were averaged.

#### Analysis of the cue-target interval

For analysis of the cue-target interval, we subjected mean power of 100-ms intervals between 200 and 1100 ms to paired-sample t-tests comparing conditions separately for the contralateral and ipsilateral or right and left hemisphere. Intervals with length of 100 ms were chosen as a balance between providing fair temporal resolution on the one hand while not ending up with a large number of statistical tests on the other hand. We only considered effects after 200 ms to exclude the time where subjects visually processed and evaluated the cue before they were able to prepare for the later button press. We accounted for multiple comparisons with the false discovery rate (FDR) method using a q-value of 0.05 [46].

#### Analysis of target-related effects

For effects following the target stimulus (prefrontal beta) mean time-frequency power in the time-window between 200–500 ms was subjected to repeated measures ANOVAs with the within-subject factors Condition (see results) and Hemisphere (left- vs. right). The time-window was based on a previous study [3], focusing on the period when participants were executing the motor response. For all statistical effects involving more than two degrees of freedom, the Greenhouse–Geisser correction was applied to correct for possible violations of the sphericity assumption [47]. We report the uncorrected degrees of freedom and the corrected probabilities.

#### Phase-coupling analysis

Additionally, we analyzed phase-coupling between electrodes as a measure of neural connectivity. We computed the phase relation between electrodes as phase-locking value (PLV) following Lachaux, Rodriguez [48]:

$${PLV}_{e1e2}=\frac{1}{N}\left|\sum exp(i[\phi_{e1}-\phi_{e2}])\right|$$

with N being the number of trials and *ϕ*_*e*1_ and *ϕ*_*e*2_ being the instantaneous phase of electrodes 1 and 2. The instantaneous phase was calculated via the Hilbert transform. We were interested in phase coupling in the cue-target interval and investigated the alpha (9–14 Hz) band, as this has been linked to top-down processing [23, 49] and inter-regional connectivity in motor control studies [27–29]. We analyzed the time-window directly preceding the target (900–1100 ms) to capture preparatory connectivity and exclude stimulus-driven effects. We investigated sensorimotor alpha coupling and computed the PLV for electrodes C3 and C4 with all other electrodes. As the PLV values were not normally distributed, condition effects were evaluated with the Wilcoxon signed rank test and corrected for multiple comparisons with the FDR method.

#### Relevant contrasts in the analysis

As measure for proactive inhibition in the cue-target interval, we contrasted trials in which the later motor response might have to be changed/inhibited (expecting change/nogo) to trials which did not require an adaptation (expecting go). To assess how proactive inhibition modulated response execution after target signals, we compared trials in which participants were prepared to execute the standard motor response (go-trials) with trials in which participants were prepared to switch to an alternative motor plan but finally had to give the standard response (no-change- and no-nogo-trials; for this account also see Swann, Tandon [12], Swann, Cai [13]). To assess correlates of reactive inhibition, we contrasted trials in which the response actually had to be changed/inhibited (change-/nogo-trials) with trials in which the standard response was given (no-change-/no-nogo-trials). For a brief summary of the contrasts used in this study see Table 1.

**Table 1:** Overview of utilized contrasts in the study.

*Cue-target interval*	
expecting change vs. expecting go	Preparation to change a standard motor response
expecting nogo vs. expecting go	Preparation to inhibit a standard motor response
expecting change vs. expecting nogo	Differential activity between preparing to change or inhibit a standard motor response
*Target-related effects*	
No-change/no-nogo vs. go	Preparation to change/inhibit a standard motor response (after target signals but proactive)
No-change/no-nogo vs. nogo/change	Implementation of a change or inhibition of a standard motor response (reactive)

The first three contrasts reflect proactive effects taking place in the cue-target interval. The latter two contrasts reflect effects taking place after target onset. Thereby the upper contrast reflects a proactive effect and the lower a reactive effect.

## Results

### Behavioral results

Reaction times differed between conditions (F_3,63_ = 119.5, p < 0.001). Participants were faster in go-trials (250±100 ms) than in no-change-trials (358±76 ms; t_21_ = 11.3, p < 0.001), no-nogo-trials (354±84 ms; t_21_ = 12.1, p < 0.001) and change-trials (403±88 ms; t_21_ = 14.2, p < 0.001) (Fig 1). Furthermore, they were faster in no-change- compared to change-trials (t_21_ = 5.4, p < 0.001) and faster in no-nogo- than in change-trials (t_21_ = 6.5, p < 0.001). There was no difference between no-change- and no-nogo-trials (t_21_ = 0.9, p = 0.382).

Accuracy was nearly at ceiling in no-change- (98%) and no-nogo- (99%) trials. It was reduced in change- (91%), go- (90%) and nogo- (87%) trials. Premature errors (button presses before the target stimulus) were committed more often in the expecting go (EG) condition (9.8%) than in expecting change (EC)- (0.45%) and expecting nogo (ES)- (0.34%) trials (F_4,84_ = 24.8, p < 0.001; EG vs. EC: t_21_ = 5.0, p < 0.001, EG vs. ES: t_21_ = 5.0, p < 0.001, EC vs. ES: t_21_ = 0.8, p = 0.432). There were more choice errors and omissions in change- vs. go-trials (choice errors: t_21_ = 4.3, p < 0.001; omissions: t_21_ = 2.4, p = 0.025). Participants failed to inhibit in 13% of nogo-trials.

### Results of the cue-target interval

#### Mu and beta power over sensorimotor cortex

**Fig 2:**
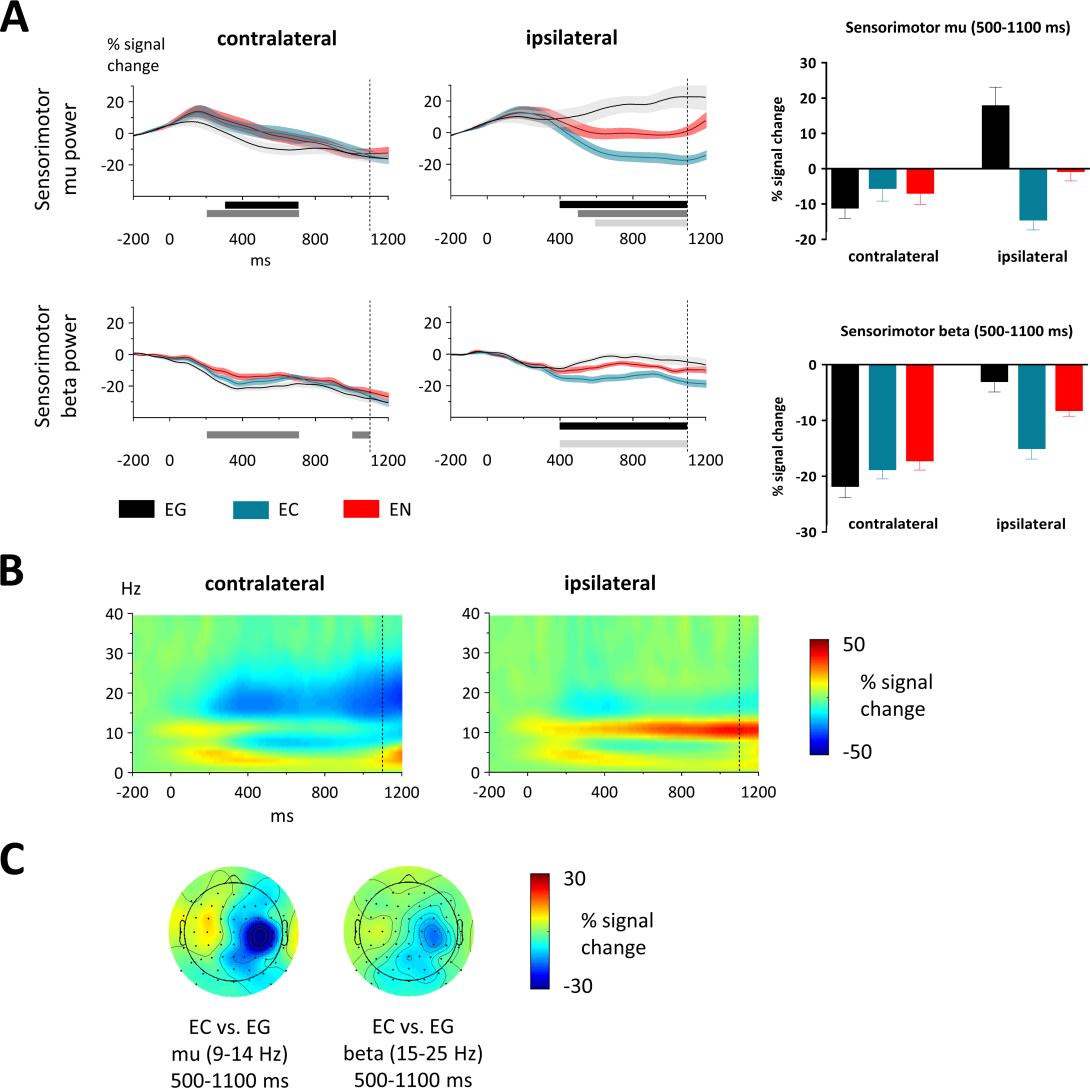
Sensorimotor effects in the cue-target interval. (A) On the left timecourses of mu (9-14 Hz) and beta (15–25 Hz) power at contralateral and ipsilateral sensorimotor clusters in relation to the standard motor response are displayed. The modulation of mu power was strongest over the ipsilateral hemisphere. Between 500–1100 ms mu increased in expecting go (EG), was around baseline in expecting nogo (EN) and decreased clearly in expecting change (EC). As shaded area around the mean, the SEM is displayed. Target onset was at 1100 ms (dotted line). Horizontal bars under the time axis highlight time-windows with significant differences between conditions. Black bars highlight windows where EG & EC significantly differed, dark grey bars where EG & EN differed and light grey bars where EN & EC differed. To the right bar graphs show mean mu/beta power between 500–1100 ms at contralateral and ipsilateral sensorimotor sites. As error bars the SEM is depicted. Significant differences are stressed with asterisks. (B) Time-frequency plots of activity in the EG condition at contra- and ipsilateral sensorimotor clusters. (C) The topographic plots show the scalp distribution of the mean signal change (500–1100 ms) as differences between EC and EG. All data in this figure is flipped along the midline.

To investigate the role of the sensorimotor cortex in proactive motor control, we analyzed mu (alpha) (9–14 Hz) and beta (15–25 Hz) activity over central sites. We subjected mean mu/beta power of 100-ms intervals between 200 and 1100 ms to paired-sample t-tests comparing different conditions separately for the contralateral and ipsilateral sensorimotor cortex to the standard motor response. Correction for multiple comparisons was applied using the FDR method. Mu was centered above bilateral motor cortex and differed between the three conditions and both sensorimotor clusters (Fig 2C). Over the contralateral motor cortex mu decreased in all three conditions constantly over the whole cue-target interval towards movement onset (Fig 2A). In an early time-interval after cue-onset, contralateral mu decreased more in expecting go- (EG) than expecting nogo- (ES) trials (200–700 ms; all t_21_ < -2.7, p < 0.01) and more in EG than expecting change (EC) (300–700 ms: all t_21_ < -2.7, p < 0.01) while there was no difference between EC and EN (all t_21_ < 0.9, p > 0.39). Over the ipsilateral motor cortex mu increased in the EG condition, remained around baseline-level in EN-trials and decreased in EC-trials. In an interval starting well before target-onset and lasting until movement onset, ipsilateral mu decreased more in EC- than EG-trials (400–1100 ms; all t_21_ < -2.5, p < 0.02) and EN- than EG-trials (500–1100 ms; all t_21_ < -2.7, p < 0.01). Mu also decreased more in EC than EN (600–1100 ms; all t_21_ < -3.1, p < 0.01).

Beta activity over the sensorimotor cortex showed generally comparable effects to the pattern in the mu band (Fig 2). Over the contralateral sensorimotor cortex, beta decreased more in EG- than EN-trials (200–700 ms, 1000–1100 ms; all t_21_ < -2.4, p < 0.03), whereas there was no difference between EC- and EG-trials (all t_21_ < 2.5, p > 0.02) nor between EC- and EN-trials (all t_21_ < 2.7, p > 0.01). Over the ipsilateral sensorimotor cortex, beta decreased more in EC than EG (400–1100 ms; all t_21_ < -3.2, p < 0.005) and more in EC than EN (400–1100 ms; all t_21_ < -2.8, p < 0.01). There was no difference between EG and EN (all t_21_ < 2.9, p > 0.008).

#### Beta power over prefrontal cortex

Mean beta power in prefrontal clusters in the right and left hemisphere between 200 and 1100 ms in intervals of 100 ms was subjected to paired-sample t-tests comparing different conditions (FDR correction was applied). Over prefrontal electrode sites, we found decreased beta power in EC- than EG-trials in the right hemisphere (500–700, 900–1000 ms: all t_21_ > 2.8, p < 0.01). There was no difference between EN- and EG-trials (all t_21_ < 2.6, p > 0.02), nor between EC- and EN-trials (all t_21_ < 2.3, p > 0.03).

#### Fronto-central alpha phase-coupling

**Fig 3:**
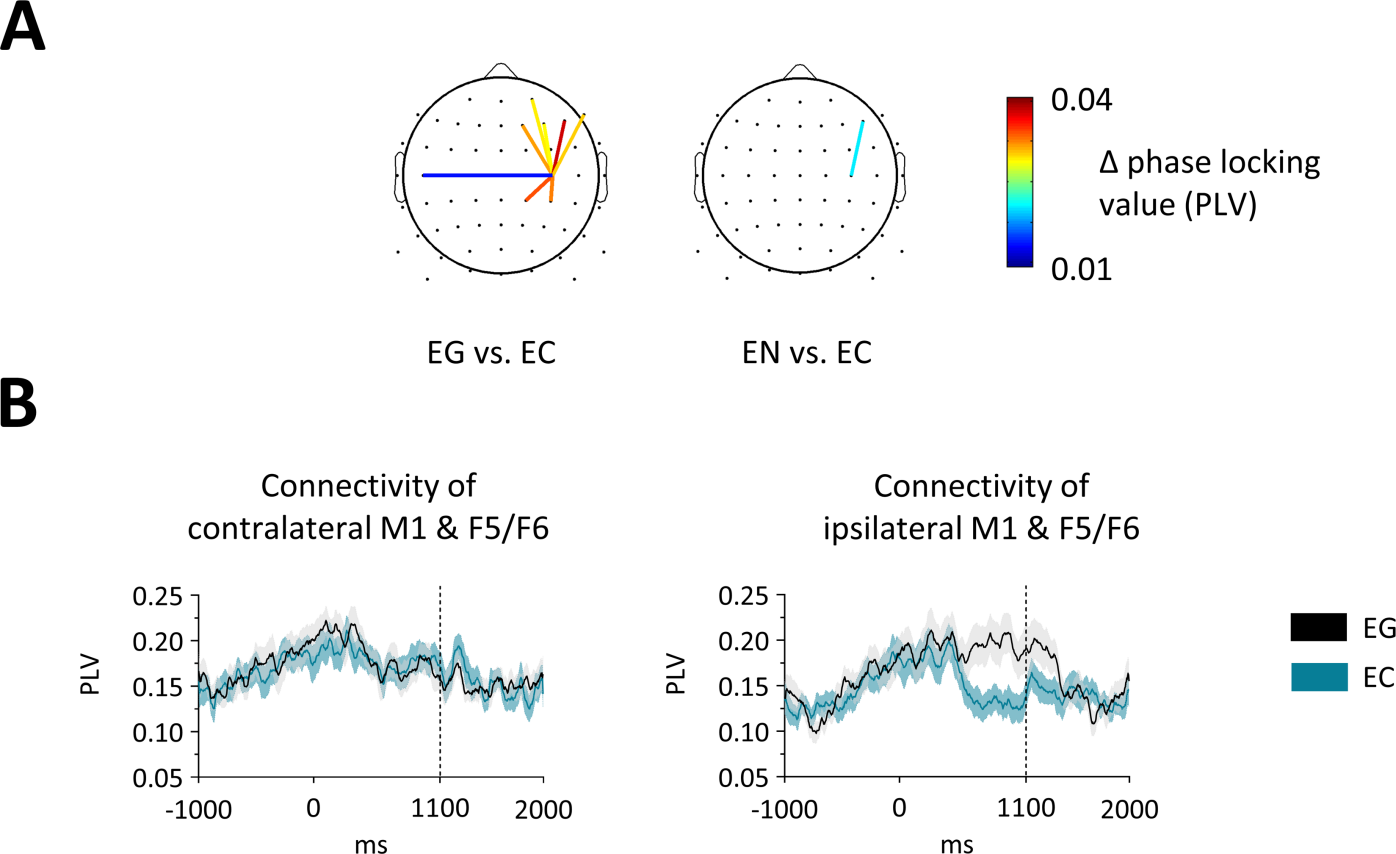
Alpha coupling effects. (A) Alpha coupling between 900–1100 ms is shown. Displayed connections show significantly increased coupling from ipsilateral motor cortex to corresponding electrodes for EG- compared to EC-trials and for EN- compared to EC-trials. (B) Exemplary time-courses of the PLV. On the left, synchrony between contralateral motor cortex and a same-side prefrontal electrode (F5/F6) and on the right synchrony between ipsilateral motor cortex and a same-side prefrontal electrode (F5/F6) is displayed. Note that there was a significant difference for connectivity to ipsilateral but not to contralateral M1.

We computed phase synchrony in the alpha (9–14 Hz) band for connections from bilateral motor sites to all other electrodes. We analyzed the time-window between 900–1100 ms, when target onset was closest and cue-related effects were most distant. We observed decreased synchrony in EC- than EG-trials (Fig 3) from ipsilateral motor cortex to prefrontal and central sites mostly in the ipsilateral hemisphere (8 connections; all z > 2.8, p < 0.045). One connection showed decreased coupling EC- compared to EN-trials (ipsilateral motor cortex to F5/F6; z = 3.5, p < 0.001). There was no effect for the contralateral side. No differences between EN- and EG-trials were found.

### Target-related results

#### Beta power over prefrontal cortex

As there is evidence for prefrontal regions to be implicated in proactive and reactive motor control after target signals, we were interested in the modulation of prefrontal beta power when implementing the change or inhibition. We subjected mean prefrontal beta activity between 200 and 500 ms to ANOVAs with the factors Condition and Hemisphere (left, right). One participant showed extreme beta power compared to other subjects (> 10 SD above mean value) in change-, nogo- and no-change-trials and therefore was removed from the statistics which are reported below.

**Fig 4:**
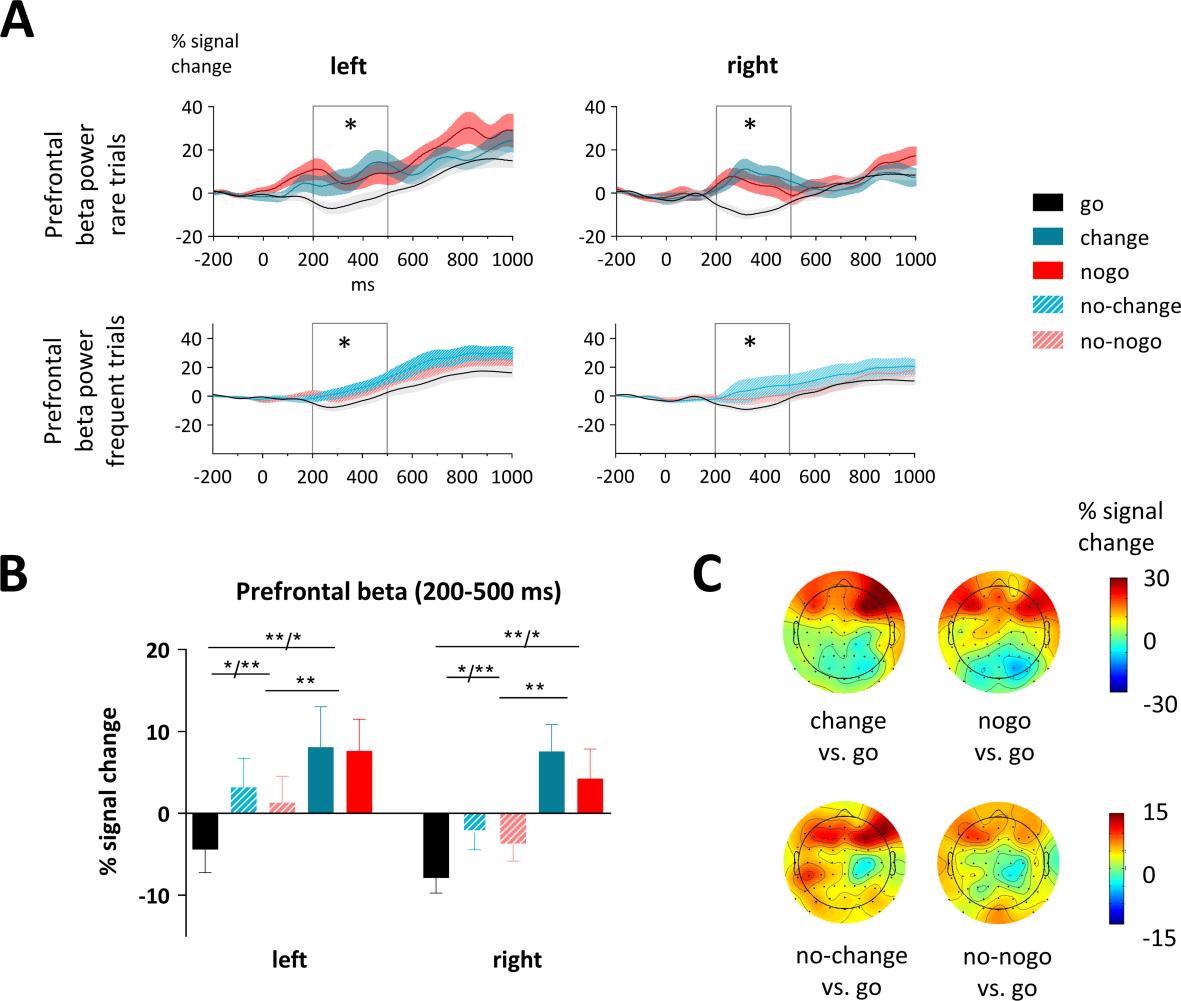
Target-evoked prefrontal beta power (15–25 Hz). (A) Timecourse of beta power at left and right prefrontal clusters. As shaded area the SEM is displayed and the time-window we analyzed (200–500 ms) is marked with a grey box. Significant differences are indicated with asterisks. (B) At both left and right prefrontal clusters beta power was higher in change- and nogo- than no-change-/no-nogo-trials and lowest in go-trials. As error bars the SEM is depicted. (C) Topographic plots show the scalp distribution of the mean signal change (200–500 ms) in the beta band as difference between conditions.

First, we were interested in whether the context of being prepared to change or inhibit a standard motor action modulates beta power over prefrontal electrodes, even if that possibility is not realized (proactive changing/inhibition after target signals). Therefore, we compared trials in which participants were cued to possibly adapt their behavior, but executed the standard response (no-change-, no-nogo-trials) with go-trials (Fig 4A, first row). Prefrontal beta was increased in no-change- and no-nogo- compared to go-trials (Condition: F_2,40_ = 9.2, p = 0.002; no-change vs. go: F_1,20_ = 11.4, p = 0.003; no-nogo vs. go: F_1,20_ = 17.5, p < 0.001; no-change vs. no-nogo: F_1,20_ = 1.0, p = 0.318). Second, we tested if prefrontal beta increases, when the standard response has to be changed or inhibited (reactive changing/inhibition) (Fig 4A, second row). Beta power was higher, when participants actually had to update their response (change/nogo), in comparison to trials where they did not have to update (no-change/no-nogo) (F_1,20_ = 9.7, p = 0.005) and in comparison to go-trials (Condition: F_2,40_ = 9.0, p = 0.001; change vs. go: F_1,20_ = 21.5, p < 0.001; nogo vs. go: F_1,20_ = 10.4, p = 0.004; change vs. nogo: F_1,20_ = 0.2, p = 0.632). As prefrontal activity has been associated with motor slowing [50, 51], we were interested if prefrontal beta power was correlated with reaction times. However, neither beta power nor beta power differences between conditions correlated significantly with reaction times or reaction time differences.

## Discussion

We investigated the oscillatory mechanisms associated with proactive motor control in a cued go-nogo-change task. In the paradigm, cues signaled participants to possibly change or inhibit a subsequent standard motor action. We found that in the cue-target interval, sensorimotor mu and beta activity differentiated a possible upcoming change from an inhibition and from a standard motor action. This modulation was strongest for sensorimotor mu ipsilateral to the hand of the standard button press. Activity in the mu band was consistent with a role of mu to selectively inhibit/disinhibit relevant neural populations (for reviews see Jensen and Mazaheri [52], Klimesch, Sauseng [53]) in preparation for upcoming behavior. During preparation to change, the ipsilateral motor cortex seems to be decoupled from prefrontal control. This was suggested by decreased alpha phase-coupling from ipsilateral prefrontal to motor cortex when participants anticipated the change. When implementing an action adaptation, beta power over prefrontal regions increased, speaking for a phasic increase of cognitive control when actually needed.

### Behavioral effects

In trials, in which participants were prepared to withhold or change their motor response (no-nogo-, no-change-), they slowed down in comparison to go-trials where inhibition was not required. This proactive slowing is in line with several other studies [3, 54–57] and facilitates action inhibition or changing when needed. Participants slowed down most when they actually had to change their motor action to an alternative response [58]. In sum, behavioral results show that participants prepared for upcoming inhibition or change of motor actions, and our experiment therefore successfully elicited proactive motor control.

### Sensorimotor activity

As the motor cortex is the final gate of motor control, it seems a likely target area to be modulated by a context in which proactive control is implemented. With this study, we provide evidence that ipsilateral sensorimotor mu and beta are modulated by the anticipation of motor adaptation and by uncertainty as to which motor action has to be performed. During the cue-target interval, mu power over the sensorimotor cortex contralateral to the standard performing hand decreased in all three conditions (expecting go/nogo/change). This is in line with previous studies which found mu to decrease in preparation of upcoming motor actions [16, 17, 19], possibly providing a sensory gating mechanism for motor preparation or anticipation of sensory input [59] and reflecting disinhibition of relevant neural populations (for reviews see Jensen and Mazaheri [52], Klimesch, Sauseng [53]). This hypothesis is supported by results from a TMS study, in which MEPs were elicited more easily when mu power over cortical motor areas immediately before stimulation was low, and vice versa [60]. The exact mechanism how mu suppression is implemented on a neural level still needs to be clarified. Some evidence points to involvement of the basal ganglia, as the degree of suppression has been linked to bradikinesia in PD patients and as the reduced ability to suppress mu in patients seems to recover with dopamine medication [61, 62]. Also, it has been suggested that prefrontal cortex modulates mu activity downstream [63]. However, further studies are needed to delineate critical nodes causing mu suppression.

In a rather early time-window (300–700 ms), contralateral mu decreased to a greater degree when subjects prepared a standard motor action (expecting go) compared to preparing both the standard action and an infrequent action (expecting change) or to cancel the response (expecting nogo). This modulation seems to reflect the higher certainty the standard motor action could be anticipated (100% in expecting go vs. 75% in expecting change/nogo), leading to a stronger disinhibition of neurons involved in movement preparation. Interestingly however, the strongest difference in mu between conditions was on the ipsilateral side. Here, mu increased when subjects prepared the standard motor action, decreased when they prepared to change and was around baseline level when they prepared to inhibit. Based on the above outlined idea of the inhibitory nature of alpha oscillations [52], the increase in expecting go-trials can be interpreted as inhibition of task-irrelevant areas, whereas the decrease in expecting change-trials might reflect the disinhibition of neural assemblies relevant for the possible upcoming change. If this was the case, mu power should be also increased in the expecting nogo condition, since similarly to the expecting go condition the ipsilateral motor cortex was irrelevant. However, this was not the case. A possible explanation is that ipsilateral mu plays an active role in motor preparation and inhibition via interhemispheric connections. Increased inhibition of the ipsilateral motor cortex in expecting go-trials might thereby facilitate action execution. Reduced inhibition as in expecting nogo-trials on the other hand would facilitate an inhibition of the contralateral side when the motor response has to be canceled later. Generally, interhemispheric inhibition seems to be important for motor control [64, 65]. Our reasoning is supported by a TMS study, which showed that left premotor cortex is involved in withholding and releasing a preselected movement generated by the right motor cortex [66]. The relevance of mu oscillations in the ipsilateral motor cortex for unimanual motor preparation and selection is further supported by a recent transcranial alternating current stimulation (tACS) study reporting reduced reaction times in a movement selection task when stimulating the ipsilateral hemisphere with 10 Hz [67]. tACS is a very novel technique, thought to entrain local brain activity in an oscillatory manner, here in the alpha band, and thereby having an impact on behavior [68]. Still, the relationship between tACS and underlying neural oscillations remains to be established. Summarizing, our findings demonstrate that the action preparation context drives ipsilateral motor cortex activity reflected in a flexible modulation of mu oscillations.

Our findings of beta power resemble those in the mu band. Importantly, similar to mu, beta was strongly modulated over ipsilateral motor cortex. Over this side however, we did not find a differentiation between expecting go- and expecting nogo-trials. Our data therefore suggests that beta reflects movement preparation less specifically than mu. A recent study found contralateral beta but not mu to reflect the directional specificity of hand movement preparation [19]. Also in our cued go-nogo task, we observed a modulation of beta power by proactive inhibition over the ipsilateral motor cortex [3]. However these two experiments differed from the present paradigm. In the former only right-handed movements were required and movement selection pertained to movement direction and not response hand as in the present study. The cued go-nogo task required frequent switches between response hands whereas response hand switches occurred in only about 8% of all trials in the current paradigm. Moreover, in another study, contralateral beta immediately before target signals was not found to be modulated by how much information about an upcoming action was presented by a preceding cue [69]. It thus remains controversial how beta reflects motor preparation and movement selection. Summarizing our sensorimotor results, the present data suggest that mu might be particularly relevant for movement selection involving both hemispheres and thus for interhemispheric connectivity.

### Sensorimotor coupling

Whereas changes in frequency-specific power at a specific region are thought to reflect local processing of information, phase coupling might represent connectivity across regions [21]. Specifically, alpha phase locking across regions is argued to reflect information transfer [70]. Based on previous findings of reactive inhibition [27–29], we hypothesized that alpha phase-coupling between prefrontal and motor cortex would be increased when subjects prepare to cancel or change actions. In contrast, we observed decreased coupling between ipsilateral prefrontal regions and the ipsilateral motor cortex when subjects prepared to change. First, this result speaks for different mechanisms taking place in proactive and reactive processes. Second, it suggests that preparing to change an action involves a relative decoupling of the ipsilateral motor cortex from prefrontal control. Also, beta power over prefrontal regions decreased when subjects prepared to change, indicating diminished cognitive control. In line with this notion, connectivity in the alpha band has been linked to top-down processing [23, 49]. A recent study reported alpha decoupling of fronto-central regions when inhibiting execution of a well-trained motor sequence and no such effect for inhibition of a novel sequence [71]. Based on this finding, the authors suggested that alpha oscillations and decoupling might serve the context-dependent inhibition of motor memory. It has also been shown that alpha coupling between mesial frontocentral areas and the primary motor cortex is reduced when novel compared to memorized sequences of complex sequential finger movements have to be executed [72]. Our findings are in line with these studies, all demonstrating a link between motor memory and fronto-central coupling. Our data extends these studies in showing that alpha/mu decoupling might not only become relevant when implementing more complex movement sequences. Instead, mu decoupling was found already when participants had to prepare for a less automatized action.

### Correlates of prefrontal control

When motor behavior needs to be adapted (e.g. changing or inhibiting it), the putative source of cognitive control modulating activity in sensorimotor regions is the PFC [73, 74]. A recent EEG study supports such a view, showing an increased negativity over PFC regions (pN), when deliberately deciding not to engage in a motor action [75]. Consistent with the notion of prefrontal beta as correlate of cognitive control [76, 77], target-related beta oscillations over prefrontal regions increased when participants had to change or cancel motor behavior. Moreover, beta increased, although to a lesser degree, when participants had been prepared to change or inhibit responses (no-change, no-nogo), but in the end did not have to. This finding suggests that prefrontal activity increases as a function of cognitive control, being higher when rare actions might have to be executed and highest when they actually have to be implemented. Importantly and in line with previous work [3, 12, 13, 56, 57], prefrontal activity increased only after target signals and not before. Moreover, as already discussed above, in the cue-target interval prefrontal beta power even decreased when participants prepared to change. In summary, this points to a twofold mechanism of cognitive control. Cognitive control is reduced to allow infrequent behavior to be prepared [72] and increases during action adaptation, possibly to implement a break over motor response tendencies [50, 51].

Our results provide evidence for prefrontal regions to be implicated in such motor braking, as corresponding to frontal beta, reaction times increased in no-change- and no-nogo- in contrast to go-trials and further increased when subjects had to change their motor action. However, we could not find any direct correlation between frontal beta and reaction times. Overall, this study adds to existing findings that frontal beta power increases not only when having to inhibit motor actions but also when implementing an infrequent action. Thus, oscillatory dynamics (here in the beta band) can be regarded as a more generalized marker of top-down frontal cognitive control [78].

### Conclusions

This study shows a task-specific modulation of sensorimotor mu/beta power in anticipation of motor action adaptation. Preparing to change or inhibit an upcoming action flexibly modulated mu power over the contralateral and especially over ipsilateral sensorimotor cortex. Moreover, alpha phase coupling between PFC and ipsilateral sensorimotor cortex was modulated proactively. This suggests that the ipsilateral motor cortex becomes decoupled from prefrontal regions when preparing to switch to an infrequent action. Prefrontal beta power increased when adapting one’s actions reflecting a phasic increase of cognitive control. Overall, our results provide evidence for a crucial role of mu oscillations in ipsilateral motor cortex and a prefrontal-motor network when preparing for action adaptation.

## Supporting information

S1 Fig Time-frequency plots. (A) Data of the cue-target interval at sensorimotor sites. Displayed are effects for the EG, EC and ES conditions separately. Note that data is flipped between hemispheres. The target appeared at 1100 ms. (B) Target-related effects at prefrontal clusters over right and left hemisphere. Here results are shown as differences between conditions.
(TIF)
